# Initial experiences and innovations in supervising community health workers for maternal, newborn, and child health in Morogoro region, Tanzania

**DOI:** 10.1186/s12960-015-0010-x

**Published:** 2015-04-09

**Authors:** Timothy Roberton, Jennifer Applegate, Amnesty E Lefevre, Idda Mosha, Chelsea M Cooper, Marissa Silverman, Isabelle Feldhaus, Joy J Chebet, Rose Mpembeni, Helen Semu, Japhet Killewo, Peter Winch, Abdullah H Baqui, Asha S George

**Affiliations:** Johns Hopkins Bloomberg School of Public Health, 615 North Wolfe Street, Baltimore, MD 21205 USA; Muhimbili University of Health and Allied Sciences, United Nations Road, 65001 Dar es Salaam, Tanzania; Jhpiego, 1615 Thames Street, Baltimore, MD 21231 USA; Ministry of Health and Social Welfare, 6 Samora Machel Avenue, 11478 Dar es Salaam, Tanzania

**Keywords:** Community health workers, Supervision, Supportive supervision, Village leaders, Tanzania, Maternal, newborn and child health, CHW, MNCH

## Abstract

**Background:**

Supervision is meant to improve the performance and motivation of community health workers (CHWs). However, most evidence on supervision relates to facility health workers. The Integrated Maternal, Newborn, and Child Health (MNCH) Program in Morogoro region, Tanzania, implemented a CHW pilot with a cascade supervision model where facility health workers were trained in supportive supervision for volunteer CHWs, supported by regional and district staff, and with village leaders to further support CHWs. We examine the initial experiences of CHWs, their supervisors, and village leaders to understand the strengths and challenges of such a supervision model for CHWs.

**Methods:**

Quantitative and qualitative data were collected concurrently from CHWs, supervisors, and village leaders. A survey was administered to 228 (96%) of the CHWs in the Integrated MNCH Program and semi-structured interviews were conducted with 15 CHWs, 8 supervisors, and 15 village leaders purposefully sampled to represent different actor perspectives from health centre catchment villages in Morogoro region. Descriptive statistics analysed the frequency and content of CHW supervision, while thematic content analysis explored CHW, supervisor, and village leader experiences with CHW supervision.

**Results:**

CHWs meet with their facility-based supervisors an average of 1.2 times per month. CHWs value supervision and appreciate the sense of legitimacy that arises when supervisors visit them in their village. Village leaders and district staff are engaged and committed to supporting CHWs. Despite these successes, facility-based supervisors visit CHWs in their village an average of only once every 2.8 months, CHWs and supervisors still see supervision primarily as an opportunity to check reports, and meetings with district staff are infrequent and not well scheduled.

**Conclusions:**

Supervision of CHWs could be strengthened by streamlining supervision protocols to focus less on report checking and more on problem solving and skills development. Facility health workers, while important for technical oversight, may not be the best mentors for certain tasks such as community relationship-building. We suggest further exploring CHW supervision innovations, such as an enhanced role for community actors, who may be more suitable to support CHWs engaged primarily in health promotion than scarce and over-worked facility health workers.

## Background

Programmes involving community health workers (CHWs) are a feature of many national health systems, contributing to reproductive health, newborn care, child survival, and prevention and treatment of chronic conditions such as HIV and tuberculosis [1-4]. While studies have shown the effectiveness of some CHW programmes, implementing these programmes at scale and in resource-constrained settings has proved difficult [3]. A common challenge concerns human resource management: how to ensure the retention, motivation, and sustained competence of CHWs, who often have limited education, operate in isolation far from health facilities, and sometimes receive only nominal pay.

One component of health programmes that is often advocated to address human resource challenges is supervision [5-12]. Supervision of facility-based health workers has received noticeable attention over the past 10 years [13]. Researchers have suggested that supervision can increase both the performance and motivation of health workers [14], although the evidence for these assertions is limited. Even less is known about the supervision of CHWs, which differs from supervision of higher-level health professionals in several ways. Compared to other front-line health workers, CHWs have less training, and CHWs operate at a distance from their supervisors, in the village, whereas facility-based health workers generally have a supervisor in place at their health facility.

The limited evidence that exists on CHW supervision suggests that facility health workers, acting as CHW supervisors, can improve the knowledge and skills of CHWs and the quality of care provided to patients [15]. Supervision of CHWs by facility health workers can raise awareness of the CHW role, legitimizing CHWs and their work in the eyes of community members [11,16]. Supervision can also bolster CHW motivation and retention [11,17-19]. Despite this growing evidence, many questions remain about the most effective supervision models and how supervision strategies can best be implemented [11]. Traditional models of CHW supervision have involved supervision from a facility health worker at regular intervals to monitor the performance of CHWs, inspect records, and correct poor practices [2,11,20]. But recently, programme implementers are testing alternative models of supervision that go beyond this paradigm.

One concept that has gained traction in the literature on facility-based health workers, and is now being applied to CHWs, is that of “supportive supervision” [21-24]. To date, few studies have examined the effectiveness of supportive supervision for CHWs. Most literature on supportive supervision involves facility-based health workers, though one recent study found that supportive supervision had a positive impact on an immunization programme involving CHWs [25]. Supportive supervision emphasizes the human aspect of supervision and involves reciprocal relationships between health workers, their supervisor, and other stakeholders. It focuses on goal-setting, identifying and resolving problems through discussions between the health worker and supervisor, promoting high standards, teamwork, and two-way communication [21]. Supportive supervision focuses more on mentoring, problem solving, and proactive planning, than on checking registers and the verification of data [24]. Quality improvement programmes in sub-Saharan Africa, including Tanzania, have suggested that supportive supervision and mentoring could help to achieve high-quality health services [26]. Following Tanzania’s health sector reform in 1999, the Ministry of Health developed an integrated health package, which included a supportive supervision component for district health management teams [27].

Another development in the supervision of CHWs is the inclusion of community members as part of a CHW’s support structure. Recent frameworks have put CHWs in the interface between the health system and the community [6,28], and increasingly policy-makers are seeing supervision as involving both health facility and community supports [6]. The involvement of community leaders has the potential to enhance community embeddedness, buy-in from community members, and community accountability. A recent study in Tanzania concluded that the involvement of village leaders in CHW supervision has the potential to increase the number of supervision contacts and improve the accountability of CHWs within the communities they serve [16].

### The Integrated MNCH Program in Morogoro region

A supervision model incorporating both supportive supervision and the involvement of community leaders was implemented in a volunteer CHW programme in the Morogoro region of Tanzania. The Integrated Maternal, Newborn, and Child Health (MNCH) Program, begun in late 2012, is an initiative of the Tanzania Ministry of Health and Social Welfare (MoHSW) and the USAID-funded Mothers and Infants, Safe, Healthy and Alive (MAISHA) programme, supported by Jhpiego. The initiative aims to improve access to and quality of MNCH services, while strengthening community and facility linkages. The MoHSW, with technical support from MAISHA, initiated the recruitment of male and female CHWs, trained for 21 days based on national MNCH CHW guidelines. CHW candidates applied, village governments nominated their top candidates, and the selection of CHWs was finalized at village meetings. CHWs were required to be residents of the village, above age 18, role models for MNCH in their community, and preferably with at least form four level of schooling. CHWs are expected to identify pregnancies, conduct routine home visits to antenatal and postpartum women and women with children up to 5 years of age, and facilitate group-based discussion sessions in the community. The topics of these discussions include antenatal care, danger signs, birth preparedness, maternal and child nutrition, postpartum and newborn care, family planning, and HIV/AIDS.

The model of CHW supervision adopted by the Integrated MNCH Program involves facility-based health workers, district and regional MoHSW staff, MAISHA staff, and village leaders from the communities in which CHWs work. The responsibilities of these stakeholders are listed in Table 1. This support structure was designed to build on the existing MoHSW cascade system of health worker supervision, wherein regional and district health management teams conduct quarterly visits to first-level health facilities. The Integrated MNCH Program sought to improve the regularity of these quarterly visits and expand their focus to include supervision of MNCH CHWs. According to programme guidelines, CHWs should receive supervision once per month from facility-based supervisors, once every 3 months (quarterly) from a delegation of district and regional MoHSW and MAISHA staff, and as often as possible on an *ad hoc* basis from village leaders [29]. CHW supervisors are service providers from the local health facility, selected based on their knowledge and experience with MNCH and willingness to serve as supervisors. The facility-based health workers that were selected to supervise CHWs were required to complete a 2-week “Community MNCH Supervisor’s Training”, which covered technical content on MNCH, supervisory roles, use of the supervision checklist, and use of reporting registers and data collection forms [29]. These facility-based supervisors are expected to practise supportive supervision of CHWs, review registers and reporting forms for data quality, discuss achievements and challenges, generate strategies to address challenges, solicit feedback from village leaders, set goals and plan activities for the upcoming implementation period, and distribute financial incentives in accordance with programme protocols. Each facility-based supervisor is responsible for supervising a total of two to four CHWs in the two villages selected for the Integrated MNCH Program in their facility’s catchment area. Although there are no explicit instructions in programme guidelines for facility-based supervisors to visit CHWs in their villages, facility-based supervisors are informally expected do this as often as possible.

**Table 1:** **Intended roles of supervisors and village leaders, adapted from programme documents** [29]

	**Facility supervisors**	**MOHSW district, regional, national teams + MAISHA**	**Village leaders**
Location	At the facility where the CHW supervisor was based (PHC or dispensary)	PHC	PHC and village
Frequency	Monthly	Quarterly	Quarterly (as part of district/regional supervision) + other ongoing informal supervision within the village
Key responsibilities	▪ Maintain a record of CHWs working in the catchment area	▪ Develop supervision standards and tools, in collaboration with key stakeholders	▪ Enable community sensitization, mobilization, and organization
	▪ Develop a monthly supervision plan	▪ Train CHWs and CHW supervisors	▪ Strengthen village health committees
	▪ Provide technical support to CHWs to facilitate community mapping and household census	▪ Provide CHW working tools and stipends	▪ Participate in selection of CHWs
	▪ Coordinate and collaborate with village government, district-level staff, and partners and develop an inventory of stakeholders within the service area	▪ Conduct quarterly meetings to discuss implementation of planned activities by CHWs, provide technical support accordingly	▪ Develop a mechanism for supporting CHWs including motivation and retention
	▪ Provide support for CHWs in planning monthly activities	▪ Collect data from facility supervisors, compile data within quarterly reports	▪ In collaboration with CHWs, organize health promotion activities in the community
	▪ Provide support to CHWs in the process of conducting household visits and other community MNCH health promotion activities	▪ Facilitate alignment or inclusion of CHW activities in Council Comprehensive Health Plans (district)	▪ Identify and discourage risky behaviours
	▪ Conduct monthly meetings to discuss implementation of planned activities by CHWs and provide technical support accordingly	▪ Facilitate availability of essential commodities and supplies	▪ Promote and support early attendance at ANC, birth preparedness, and importance of facility delivery and follow-up after delivery
	▪ Collect data from CHWs in the catchment area, compile data within monthly reports	▪ Facilitate care-seeking and provision of transport for referral	▪ Implement community management information system
	▪ Manage referrals made by CHWs to health facilities		▪ Support immunization services
	▪ Collaborate with village government in selecting CHWs		▪ Ensure registration of pregnancies, births, and deaths
			▪ Provide support for referrals to health facilities in case of emergencies
			▪ Plan and implement village health days

This paper explores the experiences of CHWs, supervisors, and village leaders involved in the Integrated MNCH Program, to understand the initial strengths and challenges of its CHW supervision model and to offer further insight into innovations that support CHWs. Our study builds on other studies conducted recently in Tanzania on CHW functioning [16,30] and supervision of facility-based health workers [31-33]. The data for this study were collected as part of a broader evaluation of the Integrated MNCH Program by the Johns Hopkins Bloomberg School of Public Health (JHSPH) and Muhimbili University of Health and Allied Sciences (MUHAS). The findings described below represent the first cycle of data collection for the evaluation and thus reflect the experiences of participants at an early stage of programme implementation.

## Methods

Quantitative and qualitative data were collected concurrently between September and October 2013. The quantitative survey was administered to 228 of the 238 MNCH CHWs reported trained by MoHSW following their recruitment, training, and deployment. CHWs trained at least 3 months prior to the start of the survey in October 2013 (from December 2012 to July 2013) were eligible for inclusion. If participants were unavailable during researchers’ first visit to a village, a return visit for the interview was arranged at a later date during the period of data collection. Participants (*n* = 10) were not included if they did not consent to the interview (*n* = 0), dropped out of the programme (*n* = 3), were travelling with unknown return date (*n* = 5), sick/hospitalized (*n* = 1), or deceased at the time of data collection (*n* = 1). The survey included modules on CHW characteristics, knowledge, training, supervision, remuneration, satisfaction, motivation, and service delivery. Indicators on supervision aimed to determine the frequency and content of supervision visits received by the CHWs. Questions on the content of supervision meetings explored activities associated with supportive supervision.

To complement data on the frequency and content of CHW supervision, we also conducted semi-structured interviews with 15 CHWs, 8 facility-based supervisors, and 15 village leaders involved with the Integrated MNCH Program (see Table 2). These interviews examined the social profile of CHWs and the interactions between CHWs, their supervisors, and village leaders. Participants were purposively sampled within specific health centre catchment areas in two districts, by their roles in the CHW programme, as well as by gender and geographical remoteness.

**Table 2:** **Summary characteristics of study participants**

	**Number**	**Male (%)**	**Female (%)**	**Mean age (years)**
Quantitative survey				
Community health workers	228	54.8	45.2	33.0
Qualitative semi-structured interviews				
Community health workers	15	60.0	40.0	31.6
Facility-based supervisors	8	37.5	62.5	41.0
Village leaders	15	93.3	6.7	44.0

Trainings for qualitative and quantitative data collection activities were conducted in parallel for both data collection teams by MUHAS and JHSPH faculty over a 1-week period (September 12 to 18, 2013). The trainings included classroom sessions on study objectives; methods, including survey design, sampling, content, and implementation; as well as ethics. Simulated interviews were conducted initially in the classroom and complimented by field-level pilot testing in Morogoro. Interviews for both quantitative and qualitative activities were conducted over a 1-hour period in the village (quantitative and qualitative) or facility (qualitative) by research assistants fluent in Swahili. All quantitative questionnaires were reviewed by the investigators for accuracy, consistency, and completeness. Quantitative data was compiled using Epi Info [34] and analysis was done using Stata 12 [35]. During qualitative interviews, research assistants took notes, which were discussed with field supervisors during daily debriefing sessions to identify emerging themes. Interviews were recorded and transcribed in Swahili and translated into English for analysis. JHSPH researchers coded and analysed the transcripts using ATLAS.ti [36]. We took a deductive approach to qualitative data analysis, examining pre-established topics of interest (such as the perceived purpose, content, and challenges of supervision), while remaining open to emerging themes.

The study was reviewed and received ethical approval from JHSPH and MUHAS Institutional Review Boards. Written consent was obtained from all study participants.

## Results

We present our findings under three topics: (1) When does supervision happen? (2) What happens during supervision encounters? (3) What happens outside of supervision encounters? These three questions are similar to those used by Marquez and Kean to distinguish between traditional supervision and supportive supervision [21]. We used this framework to assess whether, and to what extent, the supervision offered to CHWs reflects the supportive and community-embedded supervision model the Integrated MNCH Program set out to implement.

### When does supervision happen?

During quantitative interviews, CHWs were asked how long it had been since they were supervised at the health facility and in their village by their facility-based supervisor and at the health facility by district-level staff. Using these data, we calculated the mean number of supervision encounters per month, the proportion of CHWs not receiving any supervision at all since being trained, and the mean time between supervision encounters (see Table 3). We used “time since initial training” as the time period and calculated the results for CHWs who had been trained at least 4 months before being interviewed. This allowed for at least one quarterly visit to have occurred since receipt of training. Of the 132 CHWs (57.9% of 228 CHWs interviewed) who were eligible, 46 had received their first training 8 or 9 months prior to the survey, and 86 CHWs had received their first training 4 or 5 months prior to the survey.

**Table 3:** **Frequency of supervision among CHWs who were trained at least 4 months prior to the survey (** ***n*** **= 132)**

	**Mean number of supervision encounters per month**	**Proportion of CHWs who received no supervision since training**	**Among those who have received supervision, mean time (months) between supervision encounters**
Supervision encounters with facility-based supervisors at the health facility	1.24 encounters (CI: 1.12–1.35)	0.8% (CI: 0.0%–2.3%)	1.10 months (CI: 0.95–1.26)
Supervision encounters with facility-based supervisors in the CHW’s village	0.50 encounters (CI: 0.37–0.62)	25.0% (CI: 17.5%–32.5%)	2.83 months (CI: 2.41–3.26)
Supervision encounters with a delegation of district and regional MoH and MAISHA staff	0.17 encounters (CI: 0.14–0.20)	42.4% (CI: 33.9%–51.0%)	3.78 months (CI: 3.48–4.08)

The results suggest that most CHWs meet with their facility-based supervisor once per month, as expected by programme protocols. The mean number of supervision encounters per month at a health facility with facility-based supervisors was 1.24. Less than 1% of the 132 CHWs said that they had not been supervised at all by their facility-based supervisor. Supervision encounters taking place in the CHWs’ villages were less frequent. The mean number of village-based supervision visits per month was 0.5, with 25% of CHWs never visited by their facility-based supervisor in their village. For CHWs who had met with their facility-based supervisor in the village at least once, the mean time between village supervision visits was 2.83 months. Supervision with district-level staff, expected to be quarterly, was the least frequent type of supervision. The mean number of district-level supervision encounters per month was 0.17, with 42.4% of CHWs saying that they had not received any supervision from district-level staff since their initial training. Among CHWs who had received district-level supervision, the mean time between encounters was 3.78 months.

Similar questions about supervision frequency were also asked in qualitative interviews. All 15 CHWs said that they see their facility-based supervisor monthly and that these meetings typically take place on the third day of the month, at the health facility, when they travel to the health facility to submit their monthly reports. Most CHWs said that their facility-based supervisors do visit them in their village, but these encounters are not as frequent or regular as their meetings at the health facility. Some CHWs said that their supervisor comes to the village every week; some said every few months; some said that their supervisor never comes to the village.

Qualitative interviews with facility-based supervisors generated similar findings to the interviews with CHWs. All supervisors confirmed the regularity of monthly CHW supervisions at health facilities. Several supervisors admitted to not visiting CHWs in their village very often, with some supervisors saying that, despite their willingness to visit villages, they lacked the necessary time and resources.

*To be honest, I never visit them. Instead they come here regularly to submit their reports. When they bring reports, we sit together and combine them from two villages and if they have a problem they present it.* (Facility-based supervisor, female, age 45)

*I do it [visit CHWs in their village] twice per month. There are a lot of other responsibilities in this center so I ask my [fellow health workers] to help me and then [I] go to see them.* (Facility-based supervisor, female, age 43)

During qualitative interviews, CHWs were also asked about supervision with district-level staff. Most CHWs said that they had been supervised by district-level staff but that these visits were sporadic. CHWs said that district-level supervision meetings were often unscheduled and usually organized at the last minute.

*We are not involved in the timetable arrangement, so we don’t know because [our supervisors] arrange it themselves… It is very difficult to discuss it because they are the ones who make decisions on when to come; we can’t tell them when to come.* (CHW, male, age 34)

### What happens during supervision encounters?

**Figure 1:**
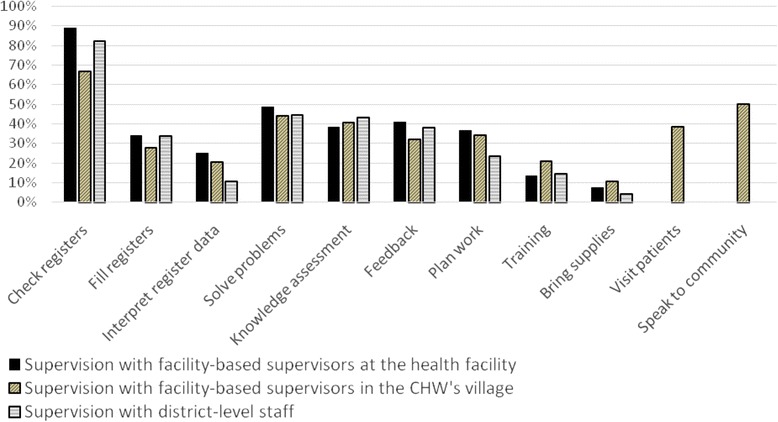
**Content of supervision, information from the quantitative survey of CHWs (** ***n*** **= 228).**

In order to understand the content of supervision, CHWs were asked during the quantitative survey to list the activities that take place during supervision encounters. As seen in Figure 1, 88.6% of CHWs said that their facility-based supervisors check their registers during monthly supervision meetings, but only 38.2% of CHWs said that their supervisions with facility-based supervisors involve a knowledge assessment, 40.9% of CHWs said that their supervisions involve feedback on work, 36.4% said that their supervisions involve work planning, and 13.2% of CHWs said that their supervisions involve training. The results are similar for supervision encounters with district staff: 81.8% of CHWs said that their district-level supervisors check their registers during supervision meetings, but only 42.9% of CHWs said that their supervisions with district staff involve a knowledge assessment, 37.7% of CHWs said that their supervisions involve feedback on work, 23.4% said that their supervisions involve work planning, and 14.3% of CHWs said that their supervisions involve training.

These findings are supported by comments made by CHWs and supervisors during qualitative interviews. When asked what the primary purpose of supervision was, the majority of both CHWs and supervisors said that the purpose was to check reports and registers. This was particularly true of monthly meetings with facility-based supervisors, which were seen as a forum for submitting reports, correcting errors in CHW registers, and replenishing supplies.

*The area I consider important is filling out the reports in their registers. You know, any work without a written record may end in vain. This is why I want them to come with their registers.* (Facility-based supervisor, female, age 42)

*I take [my register] to [the health facility], but since we all bring them on the same day it looks like a meeting. The supervisor checks and analyses the data and our plans of work then we discuss together to get everything right. So it is like a meeting for the supervisor.* (CHW, male, age 54)

Although our findings suggest that CHW supervision focuses primarily on accountability and report checking, CHWs overwhelmingly said they feel positive about supervision and appreciate the support offered by facility-based supervisors. The supervisors themselves also spoke positively about supervision as an opportunity to provide feedback and support to CHWs. CHWs most appreciate how supervision helps them improve their work. They are glad when their supervisor corrects their report-writing mistakes, as it means they can do their job better, and they are pleased to be able to ask questions during supervision to clarify the protocols they should be following.

*I am just happy about [being supervised] because when you meet with the supervisors you can be corrected or congratulated, so I am happy about it.* (CHW, female, age 24)

*What I like is when I write a monthly report and get suggestions from [my supervisors]. They show me where I went wrong and how to correct it.* (CHW, male, age 29)

Some CHWs said that they would appreciate more training. Although these CHWs were likely referring to formal training outside the context of supervision, it highlights the opportunity for training during supervision meetings.

*[The training] has enlightened me and increased my working ability and made the community to like me. I urge them to provide us with more trainings regularly; they should not get tired of doing that.* (CHW, female, age 31)

We also collected quantitative data on the content of village supervision visits. Most of the activities in facility-based supervision encounters also happen in village-based supervision encounters: 66.7% of CHWs said that their supervisors check their registers during village supervision visits, 40.3% said that village supervision visits involve a knowledge assessment, 34.0% involve work planning, 31.9% involve feedback on work, and 20.8% involve training. However, unlike facility-based supervision encounters, village supervision visits involve interaction between the supervisor and community members: 38.2% of CHWs said that during village-based supervision, their supervisor visits patients with the CHW, and 50.0% said that their supervisor speaks to community members about the CHW’s work.

From the qualitative data, it seems that the mere fact of supervisors coming to a village and meeting with CHWs in front of patients and other community members is important for CHWs. Several CHWs said that they greatly appreciate the visits their supervisor makes to their village. If community members question the advice provided by a CHW, the supervisor can affirm the skills and knowledge of the CHW, which improves the CHW’s reputation among community members. The supervisor can also help manage any difficult relationships in the village.

*The most important thing is when [my supervisor] schedules to visit community health workers. For instance, he may say, ‘Today I visit you!’ I feel confident when I get support from him because, if I make any mistakes in the way I provide health education to the community, he can correct me.* (CHW, male, age 27)

*In supervising, I sometimes go with these workers to visit households in order to provide education. What I do there is to check whether what they do is consonant with their job aids.* (Facility-based supervisor, female, age 42)

Qualitative interviewers also inquired about the role of village leaders in supervision. The relationship between facility-based supervisors and village leaders seems to cut both ways, with village leaders keeping facility-based health workers informed and health workers relaying information from CHW reports to village leaders. Village leaders themselves did not talk about their role in terms of formal supervision responsibilities or routine meetings with the CHW; rather, they talked about the role they play on an ongoing basis, working with facility-based supervisors and community members to keep CHWs accountable and ensure CHWs are doing what they are supposed to be doing.

*[My supervisors] want to be sure if we really work or we just bring them reports. One can fabricate a report. That’s why they come to the leaders to see and ask them if we visit our areas.* (CHW, male, age 34)

*We present our report to the supervisor at the center and the supervisor takes it back to the village leaders. The supervisor receives my report and my colleague’s report and compiles them. After compilation, he sends the feedback to the village leadership.* (CHW, male, age 29)

*I take their problems because I am their supervisor, because I am close with the village executive office and the chair person. If it is something urgent that we cannot wait for the village meeting then I go direct to see the village leadership and tell them the problem.* (Facility-based supervisor, male, age 49)

### What happens outside of supervision encounters?

In this section, we examine the support provided by supervisors and village leaders to CHWs outside the context of formal supervision encounters. A key characteristic of supportive supervision is that support should extend beyond face-to-face meetings. Supervisors should follow up the issues raised by CHWs, advocate for CHWs in the health system and in the community, and support CHWs in other ways as needed, not only in meetings [21,22].

This type of ongoing support was most apparent from village leaders, who did not meet routinely with CHWs for formal supervision meetings, but who nonetheless took action to support CHWs. In qualitative interviews, CHWs said that village leaders frequently helped with community relationship-building, raising the profile of CHWs in the community, and resolving conflict with community members. Village leaders welcomed and championed CHWs after the CHWs were trained and called meetings to bring attention to health issues and activities.

*After coming from trainings there, we introduced [the CHWs] to the community at the public meeting, [and we told the community] that if you see them coming to your houses, you should give them cooperation in what you will be asked or educated… We ask that there should be cooperation, so [the CHWs] become known to the community.* (Village leader, female, age 51)

*I work in this village, so the village chairperson and the committee in general should know what I am doing, because, during the training, we were introduced to the village leadership. The village leaders also held a meeting with the community members and we were also introduced to them.* (CHW, male, age 27)

*Well, I think because they are trained to serve the society, I make sure that when they want to meet people I help them to call people. This is because I know their skills are beneficial to me, and to the entire society… When they want to see the community members or have a meeting, I do help them… If they need anything, for example to meet people, we convene the meeting and they talk to people.* (Village leader, male, age 37)

*When we need a meeting we go and tell the leaders and they organize a meeting for us and we conduct the meeting.* (CHW, male, age 37)

Some CHWs said that community members sometimes do not accept their advice or are suspicious of the CHW’s activities. In these situations, village leaders assist by advocating for CHWs and resolving problems between CHWs and community members.

*The village leaders supervise me… When there is a problem in the village I take it to them, they help me to solve it. If there are villagers that refuse when I tell them to go and get services, it is like they despise me, so when you involve the leaders they go and tell them nicely and they understand.* (CHW, female, age 19)

*When I reached a certain family… I told them I am the community health worker, but they told me Mr. I don’t have time for that, so I stopped and went to the leaders. They called the family and explained that when the CHW comes again to accept her/him. We went for the second time, they received me warmly, and we are going on well.* (CHW, male, age 22)

When prompted, CHWs also talked about their sense of accountability to village leaders and the oversight they provide. This notion was echoed by village leaders themselves.

*We have a close relationship. For example, in this health service we must have a relationship with the village leaders because we cannot do anything without them knowing.* (CHW, female, age 31)

*My responsibility is checking whether [the CHWs] are working… taking their information… to the dispensary… So when these community health workers do not perform well I always report to the doctor at the dispensary so that they can be replaced or warned.* (Village leader, male, age 28)

Support from facility-based supervisors outside of supervision encounters was not as extensive as the ongoing support provided by village leaders. Some CHWs said that facility-based supervisors help with problems that arise with families in villages. Other CHWs felt that supervisors were less able to resolve problems, particularly broader health system problems. For example, many CHWs raised concerns about transport issues and the fact that they had not been given bicycles as promised, but supervisors were viewed by CHWs as not being able to do anything about this problem. Even the supervisors themselves said that there were certain issues they could not do anything about.

*The supervisor will just tell you that the problems have already been presented to the top leaders and that they [the top leaders] will solve it; but the problems still persist.* (CHW, male, age 34)

This sentiment was extended to the lack of tangible incentives offered by facility-based supervisors to CHWs, particularly the limited financial incentives. CHWs said that words of encouragement from supervisors and training opportunities for CHWs were motivating. But almost every CHW said that supervisors (or the health system in general) should offer CHWs a larger stipend than the small stipend they currently receive.

*Supervisors should motivate workers, for example maybe they plan that let’s give a certain amount so that the workers can be motivated and fulfill their responsibilities well.* (CHW, female, age 19)

Likewise, CHWs felt that the incentives offered by village leaders were limited. Village leaders said that they would like to support CHWs more, because they understand the financial difficulties CHWs encounter in carrying out their duties, but village leaders lack financial resources themselves. Some village leaders said that they exempted CHWs from village duties as an incentive, though this was not mentioned by either CHWs or facility-based supervisors.

*They have been exempted from all the minor village contributions - that is my biggest help. There were contributions, maybe for secondary, primary schools, volunteering to bring something, there is carrying bricks… those are not involved, all my people in health services don’t do it* (Village leader, male, age 61)

*We were told when we were in training that we should advise the village leaders to find a way to motivate these CHWs. For instance, we have the CHF [the community health fund] but they are not members of the fund. So they were of the opinion that the village government could do something to get them enrolled in this fund… We reported this matter to the village leaders but they have done nothing as yet. The guys [CHWs] feel as if the village government has abandoned them.* (Facility-based supervisor, female, age 42)

*My major responsibility is only to ask how they are keeping on… I was also told as the village we should give them allowances, that's my responsibility. I haven't done it yet because I am not good financially.* (Village leader, male, age 61)

## Discussion

The Integrated MNCH Program aims to integrate supervision of MNCH CHWs into an existing cascade system of supervision recommended by the MoHSW for facility-based services. This study explores the experiences of CHWs, supervisors, and village leaders regarding CHW supervision at an early stage of programme implementation. Our findings reveal many positive aspects of supervision in the Integrated MNCH Program. The programme has successfully brought a range of actors into the support structure for CHWs: facility-based health workers, staff at district and regional MoHSW offices, MAISHA staff, and village leaders from the local communities in which CHWs work. Mobilizing these people is an achievement in itself, particularly the mobilization of village leaders, whose involvement in CHW supervision is not typically sought or obtained. CHWs in the programme see their facility-based supervisors monthly, and the content of these encounters includes activities that go beyond what has traditionally been envisaged for CHW supervision; over half of the CHWs said that their meetings with facility-based supervisors involve some form of problem solving or knowledge assessment. CHWs said that they were appreciative of supervision and found it motivating. These findings suggest that the Integrated MNCH Program has made progress towards meeting its objective of supportive, community-embedded supervision for CHWs.

Our results also highlight several challenges with CHW supervision. Most supervision appears to take place at the health facility, which may facilitate linkages with facility-based services and supervisors but exact a toll on CHWs who have limited time for programme activities and service delivery. Some CHWs said that their monthly meetings with facility-based supervisors happen in a group with other CHWs, limiting the opportunities for one-on-one mentoring and individual feedback. Supervision is seen by CHWs primarily as a means for submitting and checking documentation and correcting errors in their reports. Facility-based supervisors do not often visit CHWs in their villages, and supervision visits from district and regional staff are infrequent and scheduled with little advanced notice. Some CHWs reported frustration at their supervisors’ inability to respond to particular concerns.

Despite these challenges, the Integrated MNCH Program’s approach to supervision is nonetheless appreciated by CHWs and supervisors. CHWs talked about supervision positively, especially the visits made by facility-based supervisors to their village. These visits, while not frequent, were perceived by CHWs to be beneficial for their work, facilitating connections and legitimacy in the community, and were highly motivating for the CHWs personally. This motivating aspect of facility-based supervision is especially important given the minimal financial incentives for CHWs in Tanzania. CHWs reported similarly positive feelings about their relationships with village leaders: their interactions with village leaders improved their work and their standing in the community. CHWs said that they appreciated the efforts of village leaders and facility-based supervisors to help solve problems. These results echo findings from the literature on the importance of supervision for motivation [17] and for increasing the legitimacy and effectiveness of CHWs in the eyes of other village members [37]. They also reflect findings from Tanzanian studies that highlight the potential of supportive supervision to improve the quality of CHW services [26].

Given that supportive supervision is valued by supervisors and CHWs alike, but not being fully realized, what action can the Integrated MNCH Program—and similar programmes—take to further support CHWs? As a starting point, programme managers could refine the supervision strategy and improve its implementation so that facility-based supervision embodies as many of the qualities envisioned by supportive supervision as possible. In November 2013, shortly after data collection for this study, supervision checklists for monthly and quarterly meetings were updated to facilitate a more holistic approach that goes beyond data verification, to promote discussion of challenges and achievements, knowledge and skills checks, technical support, and action planning—this is a positive step. Additional training and support for supervisors may also be necessary to shift the focus of supervision from report checking to mentoring. Behavioural and attitudinal changes must be engendered among all health workers if supportive supervision is to be effectively translated from policy into practice. The initial training of CHWs could be expanded so CHWs have more confidence and clarity around record-keeping, resulting in less need for report checking during supervision visits.

Beyond these implementation improvements, this study presents an opportunity to reflect more broadly on what we ask facility-based supervisors to do and our approach to CHW supervision in general. Perhaps the supervisors in the Integrated MNCH Program are not supervising CHWs as fully as hoped, not because the programme has not been well implemented, but because our expectations of supervisors are unrealistic. Is it reasonable to expect facility-based supervisors to meet with each of their CHWs individually every month to provide supportive supervision; to travel to each village, spend time discussing context-specific problems, and offer one-on-one mentoring? Is it realistic to expect an envoy of district-level staff to provide meaningful supervision to all the CHWs in their district every quarter? Resources to facilitate district-level supervision of CHWs are likely to be more substantial than those for supervision offered locally. Even without CHW supervision responsibilities, the demands on health facility workers are high in Tanzania and in most other low-resource settings. Human resource shortages are one of the reasons why CHW programmes are advocated, but these same human resource shortages make CHW supervision difficult. Resource factors such as fuel and transport compound the difficulties, and high health worker mobility may mean that facility-based health workers never know CHWs well enough to provide meaningful support. These issues have been raised elsewhere in studies on health worker and CHW supervision [31]. Qualitative findings from a systematic review of lay health worker programmes suggest that supervisors often lack supervisory skills and face constraints due to time and transportation [20]. A randomized control trial of an enhanced supervision programme for eye care in three sub-Saharan African countries, including Tanzania, found only modest improvements in the skills and knowledge of health workers receiving enhanced supervision, with authors concluding that the lack of programme impact may be linked to poor health system functioning and high staff turnover [32].

We might also consider the demands placed on CHWs, particularly in terms of record-keeping and reporting. What is a reasonable amount of data for CHWs to collect? Program implementers should be mindful of the unintended consequences of monitoring and evaluation (M&E) protocols that risk hijacking CHW supervision for report verification, rather than mentoring, problem solving, and skill development. M&E tools and protocols should match the skills and needs of CHWs and supervisors, and not burden supervisors or CHWs unnecessarily. As a guiding principle, facility-based supervision should focus on what is needed for CHWs to carry out their work effectively, and CHW records should focus exclusively on data that is relevant for—and actually used for—decision making and programme improvement. Implementers and researchers should consult all stakeholders, including CHWs themselves, to develop M&E and supervision models that are feasible and appropriate for the tasks CHWs are asked to undertake.

Finally, it might be worth asking whether facility-based health workers are in fact the best people to offer advice to CHWs for certain CHW activities. While clinical supervision of CHWs by health workers may be appropriate in a community case management (CCM) programme, which requires CHWs to make clinical decisions, supervision in a health promotion programme may be better offered by other people who can more easily help CHWs build trust and legitimacy in their community. Facility health workers often do not come from the same village or region as the CHWs and might not speak the local language. The power dynamics between health workers and CHWs could also play a role: supportive supervision may be more appropriate and effective for the supervision of facility health workers by district staff—who both typically have clinical training and salaried positions—than for the supervision of CHWs by facility health workers. While our study was not able to explore these dynamics, it is possible that CHWs in the Integrated MNCH Program, whose goal is health promotion for behaviour change, have certain support needs that are best met by other complementary structures in addition to the support provided by health facility workers.

One such mechanism highlighted in this study was the involvement of village leaders. The Integrated MNCH Program mobilized village leaders to be aware of and, to a certain extent, engaged in CHW activities. CHWs clearly value the support they receive from village leaders, as “enablers” to facilitate relationship-building in the community, for help with conflict management, and to bolster the respect CHWs receive from community members. In the context of a CHW program that is mainly focused on health promotion and home visits, if programme implementers were to strengthen the capacity of village leaders, and give them a more extensive and more formal role in the programme, they could fill some of the gaps that we currently expect facility-based supervisors to fill. If village leaders or other community actors are willing to further support CHWs, implementers should develop this role with the participation of the community actors themselves. There may also be opportunities for other community-based supervision mechanisms, such as CHWs working in neighbouring communities to monitor and support each other between formal supervision meetings. A study in southern Tanzania compared the frequency of supervision visits between a facility-led and community-linked supervisory approach where village leaders were introduced as additional community-based supervisors [16]. After 6 months, the study found a 50-fold increase in the number of supervision contacts in the community-linked group, including an increase in visits with facility-based supervisors for technical assistance; the authors concluded that the involvement of village leaders in CHW supervision has the potential to increase the number of supervision contacts and improve the accountability of CHWs within the communities they serve [16]. Future research should investigate these community-based mechanisms and how they could link to, and complement, existing supervision from facility-based health workers and other health system supports.

### Limitations

When we conducted this study, the Integrated MNCH Program had been in effect for less than a year. CHWs and supervisors had been trained a maximum of 9 months prior to being interviewed. Our findings therefore reflect the experiences of participants at an early stage of the programme. CHW and supervisor experiences of supervision may change in the coming years, as supervisors develop their skills and as implementing partners adjust and strengthen programme protocols. Indeed, the MoHSW and MAISHA have already revised supervision tools to facilitate more in-depth discussion of challenges and successes, skills/knowledge review, technical support, and action planning during each supervision visit.

In quantitative and qualitative interviews with CHWs, we asked about the supervision that CHWs receive from different people: from facility-based health workers and from district and regional staff. But it may be the case that CHWs do not distinguish between monthly supervision from facility-based health workers and quarterly supervision from district and regional staff. If this was the case for some CHWs, our results on these two types of supervision may represent CHWs’ perspectives on supervision from health system actors in general, rather than from specific people. The issues raised are nonetheless important and worth discussing.

## Conclusions

Supervision during the early stage of the Integrated MNCH Program was valued by CHWs and supervisors alike, but not all aspects of the supervision model were always fulfilled. Supervision of CHWs could be strengthened by streamlining supervision protocols to focus less on report checking and more on problem solving and skills development. Some challenges with CHW supervision may not be failures on the part of the programme or supervisors, but rather reflect unrealistic expectations of what facility health workers are able to achieve, given human resource shortages and social constraints. Facility health workers, while important for technical oversight, may not be the best mentors for certain tasks such as community relationship-building. We suggest exploring CHW supervision innovations that provide complementary support mechanisms, such as an enhanced role for community actors, who might fill gaps in village-based support that facility health workers are unable to provide.

## Abbreviations

CCM: Community case management
CHW: Community health worker
JHSPH: Johns Hopkins Bloomberg School of Public Health
M&E: Monitoring and evaluation
MAISHA: Mothers and Infants, Safe, Healthy and Alive
MNCH: Maternal, Newborn, and Child Health
MoHSW: Ministry of Health and Social Welfare
MUHAS: Muhimbili University of Health and Allied Sciences
USAID: United States Agency for International Development
